# Relationships Between Oral Microecosystem and Respiratory Diseases

**DOI:** 10.3389/fmolb.2021.718222

**Published:** 2022-01-04

**Authors:** Jiajia Dong, Wei Li, Qi Wang, Jiahao Chen, Yue Zu, Xuedong Zhou, Qiang Guo

**Affiliations:** ^1^ Department of Pulmonary and Critical Care Medicine, West China Hospital, Sichuan University, Chengdu, China; ^2^ State Key Laboratory of Oral Diseases, National Clinical Research Center for Oral Diseases, West China Hospital of Stomatology, Sichuan University, Chengdu, China

**Keywords:** oral microecosystem, respiratory disease, respiratory pathogen, oral microorganism, dental plaque, oral health care

## Abstract

Oral microecosystem is a very complicated ecosystem that is located in the mouth and comprises oral microbiome, diverse anatomic structures of oral cavity, saliva and interactions between oral microbiota and between oral microbiota and the host. More and more evidence from studies of epidemiology, microbiology and molecular biology is establishing a significant link between oral microecosystem and respiratory diseases. Microbiota settling down in oral microecosystem is known as the main source of lung microbiome and has been associated with the occurrence and development of respiratory diseases like pneumonia, chronic obstructive pulmonary disease, lung cancer, cystic fibrosis lung disease and asthma. In fact, it is not only indigenous oral microbes promote or directly cause respiratory infection and inflammation when inhaled into the lower respiratory tract, but also internal environment of oral microecosystem serves as a reservoir for opportunistic respiratory pathogens. Moreover, poor oral health and oral diseases caused by oral microecological dysbiosis (especially periodontal disease) are related with risk of multiple respiratory diseases. Here, we review the research status on the respiratory diseases related with oral microecosystem. Potential mechanisms on how respiratory pathogens colonize oral microecosystem and the role of indigenous oral microbes in pathogenesis of respiratory diseases are also summarized and analyzed. Given the importance of oral plaque control and oral health interventions in controlling or preventing respiratory infection and diseases, we also summarize the oral health management measures and attentions, not only for populations susceptible to respiratory infection like the elderly and hospitalized patients, but also for dentist or oral hygienists who undertake oral health care. In conclusion, the relationship between respiratory diseases and oral microecosystem has been established and supported by growing body of literature. However, etiological evidence on the role of oral microecosystem in the development of respiratory diseases is still insufficient. Further detailed studies focusing on specific mechanisms on how oral microecosystem participate in the pathogenesis of respiratory diseases could be helpful to prevent and treat respiratory diseases.

## Introduction

Oral microecosystem is a very complicated ecosystem that is located in the mouth and comprises oral microbiome, anatomic structures of oral cavity (teeth, periodontium, tongue, oral mucosa, etc.), and saliva, in which also included interactions between oral microbiota and between oral microbiota and their hosts (Wang, 2019). As exposed to air, the mouth is in direct contact with the outside environment and is constantly challenged by microbial species present in the environment via breath and eating. The complex anatomical structure of oral cavity (as well as dental repairing materials), the presence of saliva, and other physical and chemical characteristics such as pH, oxygen content and temperature in the environment determine the organisms that settle down in oral microecosystem (Kilian, 2018). The oral microecosystem of human harbors a vast amount of highly diverse microorganisms, including bacteria, fungi, protozoa, mycoplasmas and viruses. ∼700 bacterial species constitute the majority of a healthy oral microbiome and are further categorized into six major phyla by 16S rDNA profiling, viz, *Firmicutes*, *Actinobacteria*, *Proteobacteria*, *Fusobacteria*, *Bacteroidetes* and *Spirochaetes* (Verma et al., 2018). Besides, fungi (more than 100 species) also constitute the significant proportion of an oral microbiota, in which *Candida* spp. are one of the most common taxa (Seed, 2014). In oral microecosystem, microbes like bacteria and fungi attach to the tooth surface and form biofilms called dental plaques with surrounded extracellular matrix, in order to protect themselves from environmental fluctuation of oral cavity and external drug stimulations, and evade host defense mechanisms (Jhajharia et al., 2015). For the most common oral diseases, caries, the major pathogen of which is *Streptococcus mutans*, and periodontal disease, the major pathogens of which are red-complex bacteria (*Porphyromonas gingivalis, Treponema denticola and Tannerella forsythia*), dental plaques are the critical pathogenic basis (Kinane et al., 2008).

The balance of oral microecosystem not only contributes to the maintenance of oral health, but also has a potential impact on overall health. Through complicated interspecies interactions like mutualism, competition and antagonism, microbes living in oral microecosystem achieve the dynamic balance with each other, as well as with the host. However, in the condition of ecological disturbance or in occurrence of oral diseases, oral microorganisms have a chance to transfer into the circulatory system or the digestive system through oral cavity, thus affecting overall health (Hasan et al., 2014; Seedorf et al., 2014). The involvement of oral microorganisms has been observed in many diseases such as diabetes (Genco et al., 2005), bacteremia (Poveda-Roda et al., 2008), endocarditis (Parahitiyawa et al., 2009), rheumatoid arthritis (Ogrendik, 2009), atherosclerosis (Chhibber-Goel et al., 2016), preterm delivery (Teshome and Yitayeh, 2016), digestive system cancers (Seedorf et al., 2014) and other diseases.

In terms of anatomy, the oral cavity is also the gateway for the respiratory tract. In the cases of breathing with the mouth, accidentally inhaling and trachea cannula, saliva in oral microecosystem would enter the respiratory tract, and on the one hand, certain behaviors like cough could make the mucus of the respiratory tract and other substances enter the mouth, thereby achieving the mutual exchange between the oral cavity and the respiratory tract. Extensive evidence from studies of epidemiology, microbiology and molecular biology has established a significant link between oral microbes, dental plaque, oral health, oral diseases and respiratory diseases. Oral microbiota is known as the main source of lung microbiome (Bassis et al., 2015; Pragman et al., 2018; Maddi et al., 2019). Microbes indigenous to oral microecosystem are likely to be inhaled into lower respiratory tract (Tunney et al., 2008; Worlitzsch et al., 2009; Yamasaki et al., 2013; Tan et al., 2014; Coburn et al., 2015; Yan et al., 2015; Wu et al., 2017; Muhlebach MS. et al., 2018), and may infect the lung and trachea by pathogenic virulence factors or change respiratory mucosal microenvironment conducive to the colonization of respiratory pathogens. Besides, the diverse environment inside oral microecosystem could provide latent sites for the lodging of common respiratory pathogens (Sumi et al., 2007; Heo et al., 2008; Bensel et al., 2010; Tan et al., 2014; Rivas Caldas et al., 2015; To et al., 2020), which could further invade the respiratory tract and cause diseases under the condition of low host resistance. Many studies have observed an association between oral health, oral diseases and risk of respiratory diseases like pneumonia, chronic obstructive pulmonary disease (COPD) and lung cancer (Bágyi et al., 2006; Awano et al., 2008; Prasanna, 2011; Zeng et al., 2012; Yamasaki et al., 2013; Chrysanthakopoulos, 2016; Zeng et al., 2016; Qian et al., 2020). Therefore, it is pretty critical to understand the role of oral microecosystem in pathogenesis of respiratory diseases, which would contribute to the prevention and treatment of respiratory diseases.

Here, we review and analyze the research status on the respiratory diseases related with oral microecosystem and potential mechanisms on how respiratory pathogens colonize oral microecosystem and how indigenous oral microbes participate in pathogenesis of respiratory diseases. Oral health management strategies maintaining oral hygiene and oral microbiome symbiosis are also summarized to guide oral care interventions on populations susceptible to respiratory infection like the elderly and hospitalized patients and reduce their incidence of respiratory diseases.

## The Respiratory Diseases Associated With Oral Microecosystem

Increasing evidence has linked oral microecosystem, as well as oral health and oral diseases, with respiratory diseases mainly pneumonia, COPD, lung cancer, cystic fibrosis (CF) lung disease and asthma (Table 1). Primary respiratory pathogens could be found in dental plaques, periodontal pockets and saliva, suggesting that oral microecosystem is a potential source of the respiratory infection and diseases (Sumi et al., 2007; Heo et al., 2008; Bensel et al., 2010; Tan et al., 2014; Rivas Caldas et al., 2015; To et al., 2020). On the other hand, the presence of orally derived bacteria in sputum and bronchoalveolar lavage (BAL) fluid have been observed by many studies (Tunney et al., 2008; Worlitzsch et al., 2009; Yamasaki et al., 2013; Tan et al., 2014; Coburn et al., 2015; Yan et al., 2015; Wu et al., 2017; Muhlebach MS. et al., 2018). Oral diseases driven by dysbiosis of oral microecosystem, typically, periodontal disease (Bágyi et al., 2006; Awano et al., 2008; Prasanna, 2011; Zeng et al., 2012; Yamasaki et al., 2013; Chrysanthakopoulos, 2016; Zeng et al., 2016; Qian et al., 2020), and poor oral health status (Wang et al., 2009; Iinuma et al., 2015; Gaeckle et al., 2018; Zhou et al., 2020) are recognized as risk factors for the occurrence and progression of many respiratory diseases.

**TABLE 1:** The relationship of oral microbes, oral infection and disease, oral health (care) with respiratory diseases.

Respiratory diseases	Oral microbes	Oral infection and disease	Oral health (care)	Respiratory pathogens detected in oral microecosystem
Pneumonia	BAL specimens (CAP patients): oral *streptococci*, *Neisseria* spp. and anaerobes (*Prevotella* spp., *Fusobacterium* spp., *Veillonella* spp., and *Clostridium* spp.) Yamasaki et al. (2013)	Dental infection as a risk factor for pneumonia Laurence et al. (2015)	Professional oral care reduces AP risk Quagliarello et al. (2005); Chebib et al. (2018); Khadka et al. (2021)	Pneumonia pathogens in dental plaques (dependent elderly): *S. pneumoniae, H. influenzae* and *K. pneumoniae* Sumi et al. (2007)
	Saliva bacteria counts as a risk factor for AP Kikutani et al. (2015); Nishizawa et al. (2019)	Periodontal disease as a risk factor for CAP Bágyi et al. (2006); Awano et al. (2008); Yamasaki et al. (2013)	Brushing teeth helps control the pneumopathogens (*staphylococci*, Enterobacteriaceae and yeasts) in resting saliva and reduce VAP risk Cecon et al. (2010); Stonecypher (2010)	VAP pathogens in dental plaques (VAP patients): *S. aureus*, *P. aeruginosa*, *Acinetobacter* species, and enteric species Heo et al. (2008)
			An increase in oral care frequency significantly reduces the NV-HAP incidence rate Giuliano et al. (2021)	Saliva (COVID-19 patients): SARS-CoV-2 To et al. (2020)
			Preoperative oral hygiene interventions such as dental brushing and professional oral plaque control reduce incidence of POP Akutsu et al. (2010); Gonzalez-Rubio Aguilar et al. (2019); Jia et al. (2020)	
			The elderly who wear denture during sleep are more likely to have tongue and denture plaque, gum inflammation, positive culture for *C. albicans*, higher levels of circulating IL-6, and an increased risk for incident pneumonia Iinuma et al. (2015)	
CF lung disease	Sputum and BAL specimens (CF patients): oral anaerobic bacteria Tunney et al. (2008); Worlitzsch et al. (2009); Coburn et al. (2015); Muhlebach et al. (2018a)	NA	NA	Periodontal pockets (CF patients): *P. aeruginosa, S. aureus* Bensel et al. (2010)
	Lower respiratory tract is dominated by oral microbiome (CF children at age 2) Muhlebach et al. (2018b)			Saliva and subgingival plaques (CF patients): *P. aeruginosa* Rivas Caldas et al. (2015)
COPD	Tracheal aspirate specimens (COPD patients with severe acute exacerbations): *P. gingivalis, Treponema denticola. Tannerella forsythia*, *A. actinomycetemcomitans* and *Capnocytophaga sputigena* Tan et al. (2014)	Periodontal disease as a risk factor for COPD Prasanna (2011); Zeng et al. (2012); Qian et al. (2020)	Periodontal therapy reduces the frequency of the exacerbation of COPD Kucukcoskun et al. (2013); Zhou et al. (2014)	Lung pathogens in subgingival plaque (COPD patients with severe acute exacerbations): *Acinetobacter baumannii*, *K. pneumoniae*, *P. aeruginosa* and *S. pneumoniae* Tan et al. (2014)
	Lung tissues specimens (mild-moderate COPD patients): the sources of the lung tissue microbiota were 21.1% (mean) oral microbiota, 8.7% nasal microbiota, and 70.1% unknown Pragman et al. (2018)		COPD patients have significant fewer teeth, higher plaque index, poorer periodontal status and poorer oral health knowledge and behaviors, Wang et al. (2009); Gaeckle et al. (2018); Zhou et al. (2020)	
	Six genera and 15 species in subgingival plaques may be associated to COPD, especially the genera *Dysgonomonas*, *Desulfobulbus* and *Catonella* with much higher proportions (COPD patients) Wu et al. (2017)			
Asthma	A stronger shift in dental biofilm microbiome compared to healthy controls, with 14 different taxa (children with allergic asthma) Arweiler et al. (2021)	NA	NA	NA
Lung cancer	Significant changes in saliva microbiome which are indicated by the significant increase of *Capnocytophaga* and *Veillonella* (lung cancer patients with SCC or AC) Yan et al. (2015)	Periodontal disease as a risk factor for lung cancer Chrysanthakopoulos (2016); Zeng et al. (2016)	NA	NA
	Lower microbial diversity and richness of salivary microbiome (non-smoking lung cancer patients) Yang et al. (2018); Hosgood et al. (2021)			
	Salivary microbiome is related to cancer pathways, p53 signaling pathway, apoptosis and tuberculosis (non-smoking lung cancer patients) Yang et al. (2018)			

BAL, bronchoalveolar lavage; CAP, community-acquired pneumonia; AP, aspiration pneumonia; VAP, ventilator-associated pneumonia; NV-HAP, nonventilator hospital-acquired pneumonia; POP, postoperative pneumonia; COVID-19, coronavirus disease 2019; SARS-CoV-2, severe acute respiratory syndrome coronavirus 2; CF, cystic fibrosis; NA, not applicable; COPD, chronic obstructive pulmonary disease; SCC, small cell carcinoma; AC, adenocarcinoma.

### Infectious Pulmonary Diseases

#### Pneumonia

Pneumonia is a very common infection that inflames the air sacs in one or both lungs. Dental plaques are known to be a reservoir for common respiratory pathogens responsible for pneumonia (Sumi et al., 2007). Community-acquired pneumonia (CAP) occurs outside of hospitals or other health care facilities and the most commonly identified bacterial pathogens of CAP could be detected in dental plaques, including *Streptococcus pneumoniae (S. pneumoniae), Haemophilus influenzae* and *Klebsiella pneumoniae* (Sumi et al., 2007). In an etiology study of CAP, high rates of oral *streptococci* and anaerobes were found in the BAL specimens of CAP patients (Yamasaki et al., 2013). The level of periodontal disease has a significant association with the mortality of pneumonia and the anaerobic bacteria from the dental plaques of patients with periodontal disease could be causative agents of CAP (Bágyi et al., 2006; Awano et al., 2008; Yamasaki et al., 2013). Thus, it is suggested by a study that the presence of dental infections may worsen the symptoms of the patients who made a visit to the emergency departments because of pneumonia and increase the chance to be hospitalized (Laurence et al., 2015). In contrast to CAP, hospital-acquired pneumonia (HAP), which include ventilator-associated pneumonia (VAP) and healthcare-associated pneumonia (HCAP), refers in particular to pneumonia occurs in hospitals (including rehabilitation hospitals, nursing homes for the elderly, etc.). The pathogenic microbial spectrum of HAP contains gram-positive cocci, such as *Staphylococcus aureus (S. aureus)* and *S. pneumoniae*, but more gram-negative bacteria, such as *Pseudomonas aeruginosa, K. pneumoniae*, *enterococci* and *Enterobacter* spp. (Miyashita et al., 2005; Cilloniz et al., 2011; Koulenti et al., 2017; Leone et al., 2018). The increasing of dental plaque is also related to the incidence of nosocomial infections of the patients in the intensive care units (ICUs) (Fourrier et al., 1998). With the aid of the molecular methods, respiratory pathogens of VAP that are isolated from dental plaque and BAL fluid from patients in ICU are found to be genetically related (Heo et al., 2008). As a form of HCAP, aspiration pneumonia increases the mortality especially in old-age people (Feng et al., 2019). Oral bacteria are recognized as a source of infection for aspiration pneumonia and oral bacteria counts are a risk factor for aspiration pneumonia (Kikutani et al., 2015; Nishizawa et al., 2019). The observation that aspiration pneumonia occurred less in older people who received professional oral care compared with no such care, indicates the importance of oral health in reducing aspiration pneumonia risk (Quagliarello et al., 2005; Chebib et al., 2018; Khadka et al., 2021).

More and more researchers are focusing on viral pneumonia, especially in the context of the current pandemic of Coronavirus disease 2019 (COVID-19), which is caused by severe acute respiratory syndrome coronavirus 2 (SARS-CoV-2). The association of COVID-19 with oral microecosystem is becoming clear. Saliva has been shown to contain infective viral loads, indicating not only its role in SARS-CoV-2 transmission but also its diagnostic value for viral RNA testing as a more easily obtainable sample (To et al., 2020; Imai and Tanaka, 2021). The finding that angiotensin-converting enzyme II, the main host cell receptor of SARS-CoV-2, is widely expressed in salivary glands, tongue and mucosa of oral cavity (Chen et al., 2020; Xu et al., 2020), also correlates oral microecosystem with the infection and transmission of SARS-CoV-2.

#### CF Lung Disease

CF lung disease is characterized by persistent airway infection with complex microbial communities which often consist of pathogens, including *S. aureus, H. influenzae* and *P. aeruginosa,* and endogenous microorganisms typically associated with the oral cavity (Salsgiver et al., 2016; Muhlebach M. S. et al., 2018). Oral microecosystem provides the habitats for the colonization of pathogens of CF lung disease. Identical facultative and obligate anaerobic strains, including *P. aeruginosa and S. aureus*, are found in CF periodontal pockets and sputum samples, suggesting that periodontal pockets is a potential source of the respiratory infection for CF patients (Bensel et al., 2010). The study of Caldas et al. also demonstrated that the primary CF pathogen *P. aeruginosa* is detected not only in the sputum samples of CF patients, but also in their saliva and subgingival plaque samples, with clonal relatedness (Rivas Caldas et al., 2015). The presence of orally derived anaerobic bacteria in sputum and BAL fluid from CF patients have been observed by many studies (Tunney et al., 2008; Worlitzsch et al., 2009; Coburn et al., 2015; Muhlebach MS. et al., 2018). Recently, a study on succession of the CF lung microbiome in early life further revealed the role of oral bacteria on the pathophysiology of CF lung disease. It was found that CF infants had relatively sterile lower airways with a progressive shift to a microbiome dominated by aerobic and anaerobic bacteria commonly associated with the oral cavity at age two, which was correlated with a significant increase in bacterial density and inflammation, and the lung microbiome was dominated by pathogens in the majority of CF children older than four, which was associated with a further increase in inflammation and the onset of structural lung disease (Muhlebach M. S. et al., 2018). These findings suggest that oral microbes may be associated with the progression of early CF lung disease and could potentially predispose subjects to subsequent infection by pathogens (Muhlebach M. S. et al., 2018). Considering the correlation between oral bacteria and CF lung disease, the impact of oral bacteria-driven diseases especially periodontal disease, as well as the oral health management measures, on the development of CF lung disease should be further investigated.

### Obstructive Pulmonary Diseases

#### COPD

COPD is a chronic inflammatory lung disease that causes obstructed airflow from the lungs. Metagenomic analysis shows that both periodontal pathogens (the red-complex bacteria, *Aggregatibacter actinomycetemcomitans* and *Capnocytophaga sputigena*) and lung pathogens (*Acinetobacter baumannii*, *K. pneumoniae*, *P. aeruginosa* and *S. pneumoniae*) are present in subgingival plaque biofilm and tracheal aspirate of patients with severe acute exacerbations of COPD (Tan et al., 2014), indicating that not only oral microecosystem is a reservoir of respiratory pathogens but also periodontal pathogens may contribute to the pathology of COPD. The study of Wu et al. found that six genera and 15 species in subgingival plaques might be associated to COPD, among which the genera *Dysgonomonas*, *Desulfobulbus* and *Catonella* showed much higher proportions in COPD patients (Wu et al., 2017). To get rid of interference of upper airway microbes, a recent study surgically obtained the mild-moderate COPD lung tissues and found that the sources of the lung tissue microbiota were 21.1% (mean) oral microbiota, 8.7% nasal microbiota, and 70.1% unknown (Pragman et al., 2018). The association between oral disease and the incidence of COPD have been proved by many studies and periodontal disease is a significant risk factor for COPD (Prasanna, 2011; Zeng et al., 2012). A most recent study by Qian et al. provides substantial evidence that risks for COPD mortality increased significantly with increasing severity of periodontitis in the elderly (Qian et al., 2020). Moreover, the periodontal therapy could help COPD patients reduce the frequency of the exacerbation (Kucukcoskun et al., 2013; Zhou et al., 2014). The observations that compared with controls with normal pulmonary function, patients with COPD have significant fewer teeth, higher plaque index, poorer periodontal status and poorer oral health knowledge and behaviors, highlight the importance of oral health care and oral health knowledge in the prevention and treatment of COPD (Wang et al., 2009; Zhou et al., 2020).

#### Asthma

Asthma is a chronic respiratory disease involving intermittent wheezing and airway inflammation. Although there are numerous risk factors to trigger an asthma attack or exacerbate symptoms, the main risk factor is proposed to be a hereditary predisposition to allergic inflammation of the bronchial tree that develops in response to respiratory allergens (Cherkasov et al., 2019). The hygiene hypothesis contends that fewer opportunities for infections and microbial exposures contribute to the prevalence of asthma and other allergic diseases. Consistent with the hygiene hypothesis, the high serum level of immunoglobulin G (IgG) antibodies to *P. gingivalis* corresponded with the low prevalence of asthma and it was concluded that colonization of the oral cavity by bacteria and other microbes might play a protective role in the etiology of allergic disease (Arbes et al., 2006). In fact, there are only few reports regarding the relationship between oral microbes and asthma or other allergic diseases and the few clinical findings are controversially discussed (Arweiler et al., 2021). Most recently, Arweiler et al. for the first time reported the contribution of dental biofilm to allergic health or disease. Through analysis of dental biofilm microbiome by a 16s-rRNA gene-based next-generation sequencing, it was observed that children with allergic asthma showed a stronger microbial shift compared to healthy controls, with 14 different taxa, suggesting a correlation between allergic asthma and oral bacteria (Arweiler et al., 2021). Several studies report that asthmatics presented with greater risk for caries development, worse gingival health, as well as disorders of salivary flow, composition and pH (Stensson et al., 2008; Mehta et al., 2009; Arafa et al., 2017). However, there is no clear evidence clarifying the role of oral health status, oral diseases and specific oral pathogens on the pathophysiology of asthma. Thus, further in-depth studies on the association of asthma with oral microecosystem become necessary.

### Lung Cancers

Lung cancer is one of the most common types of cancer, with the leading cancer mortality worldwide. Besides chemical carcinogens, bacterial and viral infections are involved in the development of lung cancer. It has been verified that the microbial factors may participate in the tumorigenesis of the lung cancer through production of bacteriotoxins and other proinflammatory factors (Littman et al., 2004; Moghaddam et al., 2011; Kovaleva et al., 2019). The lung has a distinct microbiome which may influence the development of lung cancer and more and more evidence suggests that oral microbiome is one of the main sources of lung microbiome (Maddi et al., 2019). Yan et al. for the first time demonstrated the association of saliva microbiota with lung cancer. It was observed that the saliva microbiome in lung cancer patients with small cell carcinoma or adenocarcinoma had significant changes compared to the controls, which was indicated by the significant increase of *Capnocytophaga* and *Veillonella* (Yan et al., 2015). Variations in oral microbiome have been associated with future risk of lung cancer among never-smokers. Lower microbial diversity and richness of salivary microbiota are found in non-smoking lung cancer patients and the abundance of certain specific taxa is associated with altered risk (Yang et al., 2018; Hosgood et al., 2021). More specifically, a greater abundance of the *Bacilli* class and *Lactobacillales* order in saliva was associated with an increased risk of lung cancer, while a greater abundance of *Spirochaetia* and *Bacteroidetes* in saliva was associated with a decreased risk of lung cancer (Hosgood et al., 2021). Furthermore, functional analysis indicates that salivary microbiome in non-smoking female lung cancer patients is related to cancer pathways, p53 signaling pathway, apoptosis and tuberculosis (Yang et al., 2018). The relevance of periodontal disease with lung cancer are also reported. A meta-analysis of cohort studies showed that patients with periodontal disease were at increased risk of developing lung cancer (Zeng et al., 2016). In addition, the association of periodontal disease indices, including probing pocket depth, clinical attachment loss and bleeding on probing, with the risk of lung cancer is demonstrated (Chrysanthakopoulos, 2016). Oral care intervention may have their roles in the prevention and treatment of lung cancer, but relevant research is needed to provide evidence.

## Colonization of Respiratory Pathogens in Oral Microecosystem

Oral microecosystem is an important reservoir for respiratory pathogens. Various respiratory pathogens have been detected in dental plaques, periodontal pockets and saliva, including *S. pneumoniae*, *K. pneumoniae, S. aureus*, *P. aeruginosa*, *H. influenzae*, *Haemophilus parainfluenzae*, *Enterobacte cloacae*, *Proteus mirabilis*, *Escherichia coli, Acinetobacter species* and SARS-CoV-2 (Sumi et al., 2007; Heo et al., 2008; Bensel et al., 2010; Tan et al., 2014; Rivas Caldas et al., 2015; To et al., 2020). Colonization of the oral microecosystem by respiratory pathogens lays a foundation for subsequent invasion and infection by these pathogens in the lower respiratory tract while the body’s immune system is weakened. Both the biological characteristics of respiratory pathogens and perturbations in oral microecosystem contribute to the adaptation and colonization of respiratory pathogens in oral microecosystem.

### Adaptation of Respiratory Pathogens to Oral Environment

When exposed to invading pathogens, oral cavity releases reactive oxygen species (ROS) and nitric oxide (NO) from neutrophils and macrophages infiltrating, which are toxic metabolites to some bacteria due to the oxidative stress they elicit (Sato et al., 2008). However, pathogens such as *K. pneumoniae* and *S. aureus* develop hydrogen (H_2_) generation to against oxidative environments in the human oral cavity (Kanazuru et al., 2010). *P. aeruginosa* is found to be killed by hydrogen peroxide (H_2_O_2_)-producing oral commensal *streptococci* in a nitrite-dependent manner through the production of reactive nitrogenous species (RNS) (Scoffield and Wu, 2015). The observation that the *P. aeruginosa* strain defective in the production of nitrite reductase, which is responsible for catalyzing the reduction of nitrite to NO, showed a reduced survival rate when co-cultured with oral commensal *Streptococcus parasanguinis* (Scoffield and Wu, 2016), suggests that nitrite reductase could play an important role in the interspecies interaction with oral microbes and the survival of *P. aeruginosa* in oral microecosystem. In addition to toxic metabolites from oral cavity, common oral antibacterial agents like chlorhexidine (CHX) are also challenge for respiratory pathogens. Nevertheless, it has been observed the adaptation of clinical *K. pneumoniae* isolates to CHX exposure, which is associated with mutations in the two-component regulator *phoPQ* and a putative Tet repressor gene *(smvR)*, could lead to not only stable resistance to CHX, but also cross-resistance to colistin (Wand et al., 2017). Duman et al. further demonstrated decreased susceptibility to the complement-mediated serum killing within the CHX adapted strain of *K. pneumoniae*, as well as increased efflux pump expression (*cepA and kdeA*) (Duman et al., 2019).

Given that oral microecosystem is a complex and open environment with saliva flow and other flowing liquids from the diet, adherence is an essential step in oral colonization and subsequent respiratory infection by a respiratory pathogen. Respiratory pathogens have developed some characteristics to facilitate their adherence capacity in oral cavity. Merghni et al. found that within the twenty-one *S. aureus* strains that were isolated from the oral cavity and detected the presence of adhesins, 76.2% of strains were *icaA* and *icaD* (encoding polysaccharide intercellular adhesins) positive and 90.5% harbored both the *fnbA* and *fnbB* genes encoding fibronectin-binding proteins. Besides, the *cna* gene encoding a collagen-binding protein and *hla* gene encoding α-toxin were found in 57.2 and 52.4% of the isolates, respectively, which both play an important role in the pathogenesis of staphylococcal diseases (Merghni et al., 2015). Moreover, Wang et al. reported that *S. aureus* could activate the COX-2/PGE2 pathway in oral epithelial cell and subsequently facilitate the growth and adherence of the pathogen (Wang et al., 2017). Proteins in human saliva could modulate bacterial colonization of the oral cavity. The study of Thamadilok et al. revealed that unencapsulated *S. pneumoniae* was able to bind to low molecular-weight salivary mucin-7, presumably through a glycan-mediated mechanism which could further mediate its adherence to saliva-covered surfaces in the oral cavity (Thamadilok et al., 2016).

### Interactions of Respiratory Pathogens With Oral Microbes Facilitate Formation of Dental Plaque

Several respiratory pathogens are reported to interact with indigenous oral bacteria. Certain interspecies interactions like coaggregation would promote the formation of dental plaque biofilm. Compared with planktonically grown counterparts, microbes growing within biofilms often exhibit increased resistance to antimicrobial compounds and innate immune mechanisms. Thus, the participation of respiratory pathogens in formation of dental plaque would contribute to their oral colonization and subsequent respiratory infection. Komiyama et al. studied the interbacterial interaction between strains of *P. aeruginosa* and strains of indigenous oral bacteria, both of which were isolated from the oral cavity of CF patients, and observed coaggregation reactions between oral bacteria (*Streptococcus sanguis, Streptococcus mitis, Actinomyces naeslundii and Actinomyces viscosus*) and both the mucoid and nonmucoid variants of *P. aeruginosa* (Komiyama et al., 1987). These findings suggest that the diverse interbacterial interactions with indigenous oral bacteria may contribute to the oral colonization of *P. aeruginosa* in CF patients and affect the course and pathogenesis of CF. *S. aureus* is also reported to coaggregate with indigenous oral bacteria like *Streptococcus* spp., *A. naeslundii*, *A. viscosus, P. gingivalis and Fusobacterium nucleatum* (Kamaguchi et al., 2003). Further analysis of the interaction between *F. nucleatum* and *S. aureus* revealed that the outer-membrane adhesin RadD of *F. nucleatum* might partially participate in aggregation and increase the expression of the staphylococcal global regulator gene *sarA* (Lima et al., 2019). It is interesting to note that colonization of *S. aureus* also benefits other respiratory pathogens, since an interaction between the SpA protein secreted by *S. aureus* and the *P. aeruginosa* exopolysaccharide Psl has been identified, which causes aggregation of bacterial cells that leaves them resistant to tobramicin (Beaudoin et al., 2017). Complex communication and interaction within dental plaque biofilm also create a condition for further progression of virulence of pathogens. For example, *nanA*, a virulence determinant of *S. pneumoniae* that is important for colonization and infection, could be evolved by recombination of the pathogen with oral *streptococci* (King et al., 2005).

### Perturbation in Oral Microecosystem Favor Colonization of Respiratory Pathogens

#### Disturbance of Oral Microecology

Normal oral microbiota plays important roles in preventing the invasion of pathogenic bacteria like *P. aeruginosa*, which is showed to fail to integrate into salivary microbial community when co-cultivated in saliva medium and whose growth is inhibited by the oral microflora via producing lactic acid (He et al., 2011). However, disturbance of oral microecology gives opportunistic respiratory pathogens chances to invade and colonize. It has widely reported that oral microbial dysbiosis or changes in oral microecosystem, such as the progression of periodontitis and denture stomatitis or the presence of dentures, promote oral colonization by *S. aureus, P. aeruginosa*, *Enterococcus faecalis* and *Acinetobacter* spp. and may favor the spread of more pathogenic strains (Baena-Monroy et al., 2005; Passariello et al., 2012; Colombo et al., 2013; Souto et al., 2014). Moreover, smoking is found to benefit early acquisition and colonization of respiratory pathogens including *Haemophilus* and *Pseudomonas* in dental plaque biofilms (Kumar et al., 2011).

One potential mechanism is that in the condition of oral microflora dysbiosis, the increase of some oral pathogens enhance their interaction with certain respiratory pathogens, as aforementioned, and further facilitate the oral colonization of these non-indigenous pathogens. Another mechanism could refer to the decrease of oral commensal bacteria, the function of which is to inhibit the invasion and colonization of external microorganism as well as to maintain balance of oral microecology (Tada and Hanada, 2010). Plenty of oral commensal bacteria are found to exhibit antagonistic effects against respiratory pathogens. The majority of oral *lactobacilli* strains are reported to suppress the growth of common respiratory pathogens including *S. pneumoniae*, *P. aeruginosa,* as well as clinical isolates of Methicillin-resistant *staphylococcus aureus* (MRSA) (Busarcevic et al., 2008; Sikorska and Smoragiewicz, 2013; Alexandre et al., 2014; Mahdi et al., 2019). Take *Lactobacillus salivarius* for example, studies revealed that it could produce LS1 against the growth of *S. aureus* and *S. pneumoniae*, and salivaricin LHM against the activity of *P. aeruginosa* (Busarcevic et al., 2008; Mahdi et al., 2019). *Streptococci* are another main source of oral commensal bacteria to antagonize respiratory pathogens, particularly *Streptococcus salivarius,* which produces a variety of bacteriocins antagonizing *S. pneumoniae* (Tagg, 2004) and *Streptococcus pyogenes* (Walls et al., 2003; Tagg, 2009; Wescombe et al., 2009; Santagati et al., 2012), and block the adherence sites to reduce *S. pneumoniae* colonization (Manning et al., 2016).

#### Impairment of Oral Immunity

Oral immune system functions as the foremost barrier and defense against pathogens, which depends on efficient function of oral mucosa, salivary glands and saliva, and gingival crevice. Saliva plays a key role in host defense against invading respiratory pathogens via antibody production (Aanaes et al., 2013; Mauch et al., 2017), blocking oral adherence (Lerrer et al., 2005), bacteriostatic or bactericidal activities (Geetha et al., 2003; Prokopovic et al., 2014), interfering with their interactions with oral bacteria (Komiyama and Gibbons, 1984). Internal and external factors such as aging, dysplasia, disease progression and treatment which influence salivary function or oral immunity have potential impacts on oral colonization of respiratory pathogens. Oral radiotherapy and chemotherapy, except for direct mucosal damage, both lead to decreased salivary flow and increased colonization of opportunistic pathogens such as *S. aureus* and *Candida* (Main et al., 1984; Jain et al., 2016). Natural aging, parotid and submandibular salivary gland aplasia or agenesis, medication (including anticholinergic drugs, diuretics, alpha-adrenergic agents, and antihypertensive agents) are able to change the saliva composition or affect the secretion or flow rate of saliva, and subsequently result in dry mouth and poor oral hygiene (Mathews et al., 2008), which may cause shifts form the normal oral microflora to a community that harbors a higher number of pathogens (Scannapieco, 1999).

What is noteworthy is that immunocompromise and general immunodepression also raise the risk of oral colonization by potential respiratory pathogens. Higher level of *S. aureus* in oral cavity of the elderly and institutionalized individuals was reported, including hospitalized and nursing home patients (Scannapieco et al., 1992; Tada and Hanada, 2010). Diaz et al. also found a higher prevalence of potentially opportunistic *Gammaproteobacteria* including *K. pneumoniae*, *Pseudomonas fluorescens*, *Acinetobacter* species, *Vibrio* species, *Enterobacteriaceae* species, *Staphylococcus* species, and *Enterococcus* species in the salivary bacterial microbiome of transplant recipients who were constantly on pharmacological immunosuppression (Diaz et al., 2013).

## Participation of Indigenous Oral Microbes in Pathogenesis of Respiratory Diseases

The presence of oral microbes with high detectable rates in sputum and BAL specimens of patients with multiple respiratory diseases suggests a potential association of oral microbes with respiratory diseases, including oral *streptococci*, *Neisseria* spp. and anaerobes (*P. gingivalis, Treponema denticola. Tannerella forsythia*, *A. actinomycetemcomitans, Capnocytophaga sputigena, Prevotella* spp., *Fusobacterium* spp., *Veillonella* spp., and *Clostridium* spp.) (Tunney et al., 2008; Worlitzsch et al., 2009; Yamasaki et al., 2013; Tan et al., 2014; Coburn et al., 2015; Yan et al., 2015; Wu et al., 2017; Muhlebach MS. et al., 2018) In fact, common oral microbes such as *P. gingivalis, F. nucleatum, A. actinomycetemcomitans and* oral *streptococci* have been reported to be involved in the pathogenesis of respiratory diseases (Petelin et al., 2004; Pan et al., 2009; Nagaoka et al., 2013; Yamasaki et al., 2013; Whiley et al., 2014; Hayata et al., 2019). Various mechanisms have been developed to help explain how indigenous oral microorganisms may participate in the occurrence and development of respiratory diseases, based on the understanding of their virulence factors or their effects on pathogenicity of respiratory pathogens.

### Oral Microbes Inhaled Into the Lower Respiratory Tract Cause Infection and Regulate Immune Responses

**FIGURE 1:**
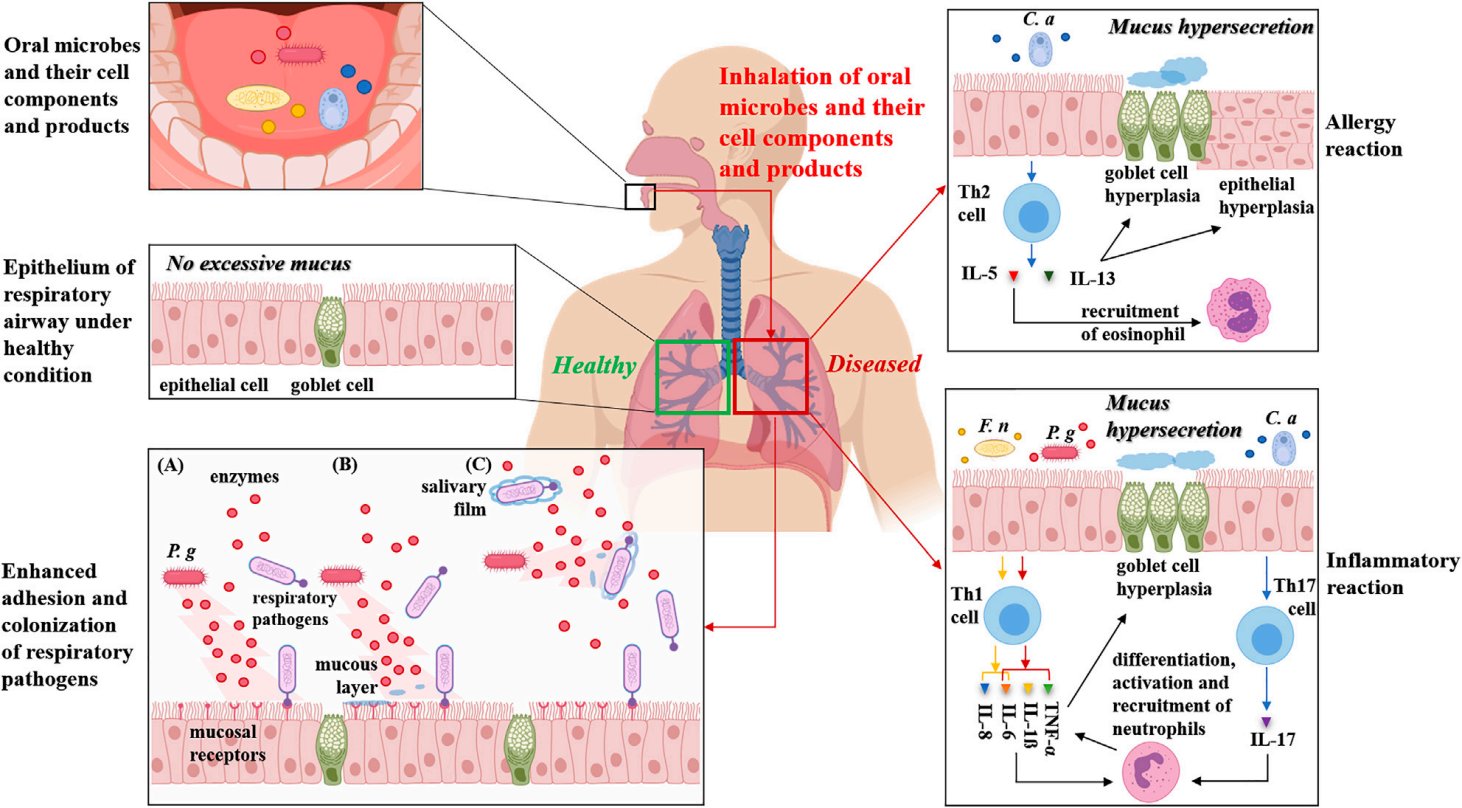
Potential mechanisms on aspiration of oral microorganisms and their cell components and products into the lower respiratory tract to modulate immunoreaction and facilitate adherence and colonization of respiratory pathogens. Indigenous oral microorganisms, as well as their cell components and products such as lipopolysaccharide, peptidoglycans, enzymes and toxins in saliva, may be inhaled into the lower respiratory tract including the lung. Here, we classify the reactions of the lower respiratory tract to invaded oral microbes and their cell components and products into allergy and inflammation. In allergy reaction mode, take *C. albicans* (*C. a*) for example, the fungus and its lysates are reported to induce IL-5 and IL-13 production by Th2 cells. IL-5 activates eosinophils which is related with airway eosinophilic inflammation. IL-13 induces goblet cell hyperplasia resulting in increased mucus production and smooth muscle cell hyperplasia and hyperactivity which are prominent pathological features of allergic reaction. In inflammatory reaction mode, typical oral microbes such as *F. nucleatum* (*F. n*) and *P. gingivalis* (*P. g*) could induce IL-8 and IL-6 production, or soluble TNF receptors, TNF-α, IL-1ß and IL-6 production, respectively, both contributing to inflammation because of the subsequent differentiation, activation and recruitment of neutrophils. TNF-α also induce goblet cell hyperplasia and mucus hypersecretion. Moreover, *C. albicans* (*C. a*) infection could induce Th17 CD4^+^ occurrence, whose main functions are the differentiation, activation and recruitment of neutrophils, which contribute to the inflammation and destruction of the lung tissue. In addition of effects on immunoreaction and inflammation, inhaled cell components and products of oral microbes (for example, *P. gingivalis*) may enhance adherence and colonization of respiratory pathogens on respiratory mucosa, mainly via three ways: **(A)** modification or upregulating expression of the mucosal epithelium receptors by specific enzymes or by other cell components and products. **(B)** clearance of the mucous layer that covers the receptors resulting in exposure of surface receptors. **(C)** the salivary film that protects against the colonization of pathogenic bacteria is destroyed by hydrolytic enzymes.

Oral microbiome is one of the primary sources of respiratory infection and indigenous oral microbes in dental plaques, periodontal pockets or saliva could be inhaled into the lower respiratory tract to cause or aggravate respiratory infections and diseases such as aspiration pneumonia and COPD. The pathogenicity of oral microorganisms invading the lower respiratory tract is involved in different mechanisms, within which the critical one is the immunoregulation on respiratory system, including T-helper 1 (Th1) and T-helper 2 (Th2) immune responses. Regulation of the Th1/Th2 balance is a typical way by which infections of microbes influence the host immune response (Schaub et al., 2006). Effects of oral microbes on inflammatory process have been widely reported, which is characterized by Th1 immune response. The data of Scannapieco et al. revealed the potential effect of oral bacteria on inducing the release of proinflammatory cytokines from epithelial cell lines to an extent similar to that seen for respiratory pathogens (Scannapieco et al., 2001). Significantly increased production of soluble TNF receptors, TNF-α, IL-1β and IL-6 are also observed in *P. gingivalis*-infected pneumonia model (Petelin et al., 2004) (Figure 1). Recently, it was showed that some periodontopathic bacteria, especially *F. nucleatum*, strongly induced IL-6 and IL-8 production by bronchial epithelial cells, which might trigger exacerbation of COPD (Hayata et al., 2019) (Figure 1).

In addition of inflammatory reactions, oral microbes are observed to impact allergy reactions. The first direct evidence on regulatory effects of oral pathogens on allergic airway inflammation and responsiveness was reported by Card et al. Their study underlined the temporal importance of the establishment of infection since *P. gingivalis* infection established before ovalbumin (OVA) sensitization reduced airway levels of Th2 cytokines (IL-4, IL-5, IL-13) and granulocyte-macrophage colony-stimulating factor, and decreased histological inflammation, without the alteration of serum levels of OVA-specific immunoglobulin E (IgE) and airway responsiveness. Conversely, a subcutaneous infection with *P. gingivalis* initiated after allergen sensitization did not alter inflammatory end points but did reduce airway responsiveness in spite of increased serum IgE levels (Card et al., 2010). Card et al. observed a reduction of the inflammatory migrated cells in the BAL fluid (eosinophils, lymphocytes, macrophages), as well as the levels of the IL-4 and tumor necrosis factor (TNF)-α cytokines, in mice with asthma and periodontitis, accompanied by a decreased mucus production (Card et al., 2010). Oral microbes also have the capacity to induce Th2 immune responses. Some studies reported that *Candida albicans*, an opportunistic pathogen that colonizes oral cavity, and its extract were able to induce IL-5 production by Th2 cells which is related with eosinophilic airway inflammation (Kimura et al., 1999; Mori et al., 2001) (Figure 1).

Apoptosis, which plays an important role in immune response and tumorigenesis, is also proposed to be affected by oral bacteria. Potential regulation of lung cancer cell apoptosis by oral microbiome through p53 pathway is observed (Yang et al., 2018). Other possible mechanisms, for example, infertile defense function of tracheal mucosa causing by the invasion of oral bacteria to surface protein (fibronectin, etc.), still need exploration and evidence (Woods, 1987; Mamani et al., 2012).

### Aspiration of Oral Microbial Components and Products Alters Respiratory Mucosal Surfaces and Modulates Immune Responses

Enzymes, cytokines and other biologically active substances in saliva which are released by oral microorganisms and inflammatory periodontal tissues could be inhaled into the lower respiratory tract and potentially influence respiratory tract mucosal epithelium. In the human airway, proteases released by invading pathogens have been associated with the regulation of the airway surface liquid layer, host defense, pathogenic infection and inflammation (Thibodeau and Butterworth, 2013). Besides proteases, it is possible for specific enzymes such as mannosidase, fucosidase, hexosaminidase and sialidase released from oral microecosystem to alter respiratory tract mucosal surfaces via modification of the mucosal epithelium or exposure of adhesion receptors located on mucosal surfaces, which would contribute to adherence and colonization by respiratory pathogens and consequent respiratory infection (Scannapieco, 1999; Gomes-Filho et al., 2010) (Figure 1). Moreover, hydrolytic enzymes from periodontal pathogens are proposed to destroy the salivary film that protects against pathogenic bacteria and leave respiratory pathogens free to enter respiratory tract, which would benefit their adherence to mucosal receptors (Scannapieco, 1999; Gomes-Filho et al., 2010) (Figure 1). Through oropharyngeal aspiration, other cell components and products of oral microbes, such as lipopolysaccharide (LPS), peptidoglycans, lipoteichoic acids, fimbriae and toxins, as well as the cytokines released from inflammatory periodontal tissues, may also alter the respiratory epithelium and promote colonization by respiratory pathogens via the upregulation of adhesion receptor expression on the mucosal surfaces or stimulate cytokine production from respiratory epithelial cells, resulting in recruitment of inflammatory cells and inflamed epithelium that is more susceptible to respiratory infection (Scannapieco et al., 2001; Gomes-Filho et al., 2010) (Figure 1).

Effects of oral microbial components and products on modulating immune responses have been revealed by many studies. Take *C. albicans* for example, oropharyngeal aspiration of fungal lysates from *C. albicans* promotes the Th2 immune response, including airway eosinophilia, secretion of Th2 cytokines and mucus cell metaplasia (Allard et al., 2009) (Figure 1). Interestingly, oropharyngeal aspiration of *C. albicans* lysates together with *P. aeruginosa* is found to result in the shift of immune response from Th2 to Th1 in an LPS/TLR4 independent but MyD88 dependent mechanism (Allard et al., 2009). Furthermore, a soluble cell-wall β-glucan from *C. albicans* could facilitate OVA-induced allergic airway inflammation in mice (Inoue et al., 2009a) and induce apoptosis and oxidative stress in the lung that enhance lung inflammation and injury (Inoue et al., 2009b).

As a hallmark of chronic airway diseases including asthma, CF and COPD, mucous hypersecretion is also related with oral microbial products. Nagaoka et al. found the products of *F. nucleatum* inhibited mucus production in high concentrations, while increased mucus production in low concentrations. Thus, aspiration of saliva containing low concentrations of *F. nucleatum* products, which is more common, might promote mucus hypersecretion in the related diseases (Nagaoka et al., 2013).

### Interactions Between Oral Microbes and Respiratory Pathogens Strengthen Respiratory Pathogenicity

**FIGURE 2:**
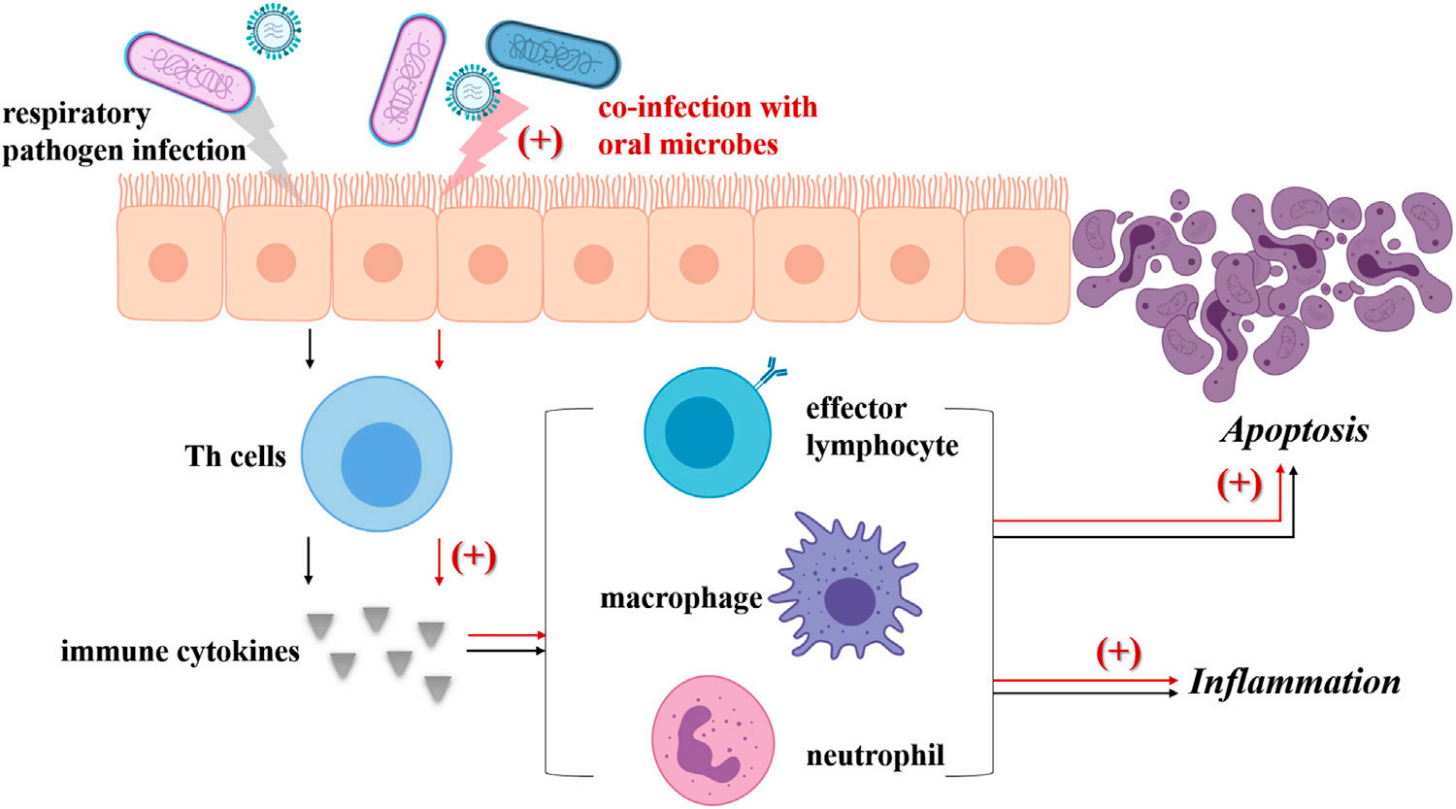
Co-infection with oral microbes strengths the pathogenicity of respiratory pathogens. Usually, respiratory pathogen infection (black arrow) could induce the Th cells to produce cytokines, then lead to the differentiation, activation and recruitment of effector lymphocytes, neutrophils and macrophages. These immune cells not only bring about inflammation, but also activate the death receptor apoptotic pathway and finally lead to apoptosis. Once oral microbes (including oral *streptococci*, *P. gingivalis*, *F. nucleatum* and *A. actinomycetemcomitans*, *Prevotella intermedia*, *C. albicans*) inhaled into airways co-infect respiratory epithelium with respiratory pathogens (red arrow), the levels of multiple cytokines are elevated including TNF-α, IL-1ß, IL-6, as well as those signaling pathway related with apoptosis of respiratory epithelial cells. Besides, some oral microbes can promote the virulence of respiratory pathogens, for example, via increasing the virulence factor production or forming pathogenic partnerships. As a result, aggravated inflammation and expanded apoptosis is observed.

Indigenous oral microbes are likely to be inhaled into the lower respiratory tract and may interact with respiratory pathogens, further modulating the pathogenicity of respiratory pathogens or both (The main mechanisms are summarized in Figure 2). Previous studies have reported the potential interactions between oral *streptococci* and *P. aeruginosa* which result in increased virulence and strengthened pathopoiesis of the latter (Duan et al., 2003; Parkins et al., 2008; Sibley et al., 2008). Recently, Whiley et al. demonstrated that pathogenic potential of the high-virulence *P. aeruginosa* CF Liverpool Epidemic Strain (LES) could be enhanced by the presence of anginosus-group of *streptococci* (AGS) and some other viridans *streptococci* (*S. mitis*, *Streptococcus oralis*, *Streptococcus gordonii* and *Streptococcus sanguinis*), and the pathogenic partnerships between the LES and AGS were verified in the *Galleria mellonella* infection model (Whiley et al., 2014; Whiley et al., 2015). However, it should be noticed that they also pointed out the negative modulation of these oral *streptococci* to *P. aeruginosa* virulence, which was found to be dependent on inoculation sequence and environment. Instead of air cooperation within a high cell density co-culture biofilm, when co-cultured in an atmosphere containing added CO_2_, *S. oralis* antagonized *P. aeruginosa* LES growth via H_2_O_2_ production. *S. mitis, S. gordonii* and *S. sanguinis* showed similar H_2_O_2_ mediated inhibitory effect when inoculated as a primary colonizer prior to introduction of the LES (Whiley et al., 2015).

There is evidence showing that co-infection of multiple periodontal pathogens and respiratory pathogens in lung epithelial cells promote the production of inflammatory cytokines and apoptosis (Pan et al., 2009; Li et al., 2014; Chen et al., 2018; Li et al., 2018). *P. gingivalis, F. nucleatum*, and *A. actinomycetemcomitans* are reported to foster *P. aeruginosa* invasion into HEp-2 respiratory epithelial cells and enhance host cell cytokine release and apoptosis (Pan et al., 2009). Further study showed that *P. gingivalis* modulated *P. aeruginosa*-induced apoptosis of respiratory epithelial cells through the signal transducer and activator of transcription 3 (STAT3) signaling pathway and their co-invasion led to greater cell death compared with *P. aeruginosa* challenge alone (Li et al., 2014). Recently, the study by Chen et al. revealed that co-infection of *P. gingivalis* and influenza A virus (IAV) subtype H1N1 in lung epithelial cells could induce the production of inflammatory cytokines including TNF-α, IL-1β and IL-6, as well as the production of NO, consequently promoting apoptosis in lung epithelial cells via the Bcl-2/Bax/caspase-3 signaling pathway (Chen et al., 2018). It was also observed that infection by *P. gingivalis* combined with IAV temporarily inhibited apoptosis in respiratory epithelial cells, possibly related to the initiation of autophagy (Li et al., 2018). Besides, the supernatant of *Prevotella intermedia* was found to promote pneumococcal adherence to human alveolar cells, elevate inflammatory cytokine levels in the BAL fluid, and increase platelet-activating factor receptor transcription (Nagaoka et al., 2014).

## Oral Health Management Strategies for Controlling Respiratory Diseases

The relationships between oral microecosystem, oral health and respiratory diseases highlight the importance of oral health management in prevention and treatment of respiratory diseases (especially pneumonia and COPD). Effective oral self-health management as well as professional oral health care, not only improve oral health, but also benefit controlling the progression or occurrence of respiratory diseases. Currently, it is clear that oral care measures maintaining oral health and oral microbiome symbiosis could reduce the incidence of pneumonia and COPD (Table 1).

### Professional Oral Health Care

Providing oral health care, including tooth, tongues and dentures, is helpful to reduce the number of microorganisms inhalable in the lower respiratory tract and control subsequent respiratory infections (Scannapieco and Shay, 2014). Professional oral health care by dentist or oral hygienists significantly benefits the populations susceptible to respiratory infection, particularly the elderly and the comatose patients. Oral health condition has been recognized as one important risk factor in the incidence of aspiration pneumonia (Quagliarello et al., 2005; Chebib et al., 2018). A systematic review conducted by Khadka et al. analyzed the articles addressing aspiration pneumonia, oral microorganisms, oral health and treatment and their results showed that professional oral hygiene care that reduces bacteria is useful in reducing aspiration pneumonia risk (Khadka et al., 2021). For nursing home residents, a weekly professional oral care leads to a significantly lower annual prevalence of pneumonia hospitalization, especially in residents whose salivary bacterial concentration exceeded the median (Chiang et al., 2020). Brushing teeth is found to be an effective way to control the pneumopathogens (*staphylococci*, Enterobacteriaceae and yeasts) in resting saliva for reducing pulmonary infection of comatose patients and brushing twice-a-day can reduce respiratory tract infections by 69 percent in VAP (Cecon et al., 2010; Stonecypher, 2010). Most recently, a Four-Unit Cluster Randomized Study assessing the effectiveness of a standardized oral care protocol in preventing nonventilator hospital-acquired pneumonia (NV-HAP) in the acute care setting found that an increase in oral care frequency significantly reduced the NV-HAP incidence rate, suggesting that daily oral care can play a crucial role in NV-HAP prevention (Giuliano et al., 2021). Many studies also reported that preoperative oral hygiene interventions such as dental brushing and professional oral plaque control are associated with a lower incidence of postoperative pneumonia (POP) (Akutsu et al., 2010; Gonzalez-Rubio Aguilar et al., 2019; Jia et al., 2020). Therefore, oral health management is a favorable factor for preventing POP, and it could be carried out before surgery. In addition, professional oral cleansing once a week for 6 months during winter could reduce influenza infection in the elderly (Okuda et al., 2005).

Based on the current understanding of the relationship between oral microecosystem, oral health and aspiration pneumonia, it has been recommended by some scholars to use oral bacterial counts and the Oral Health Assessment Tool which assesses oral hygiene as a tool to assess the requirement of taking oral care and other preventive procedures in patients at high risk of aspiration pneumonia (Nishizawa et al., 2019). Such an oral health and oral hygiene assessment tool could be developed into a standardized oral assessment procedure and applied in the treatment of respiratory diseases related to oral bacteria and oral health.

### Personal Oral Health Care

Common oral care, such as tooth brushing, flossing and regular dental visits, has been shown to be beneficial to controlling respiratory diseases. Through comparing 20 COPD individuals with 10 healthy individuals as control, Gaeckle et al. found that the healthy controls flossed frequently as their regular dental care while COPD patients not, and the control group also visited dental clinic more regularly (Gaeckle et al., 2018). Further analysis on the observation that COPD patients have fewer teeth and higher plaque index than the controls with normal pulmonary function revealed that inappropriate tooth brushing method, lower regular supra-gingival scaling and poorer oral health knowledge were significantly associated with the risk of COPD, indicating the importance of promoting dental care and oral health knowledge that can be integrated into the prevention and treatment of COPD (Wang et al., 2009). Oral health behaviors are also related to the incidence of pneumonia. It was found that denture wearing during sleep would increase the risk of incident pneumonia in the elderly and the elderly having this behavior were more likely to have tongue and denture plaque, gum inflammation, positive culture for *C. albicans*, and higher levels of circulating IL-6 as compared with those who remove their dentures at night, suggesting potential implications of oral hygiene programs for pneumonia prevention in the community (Iinuma et al., 2015).

### Utilization of Antimicrobial Agents and Probiotics

There has been evidence showing that mechanical oral care combined with povidone iodine (PVP-I) significantly reduced the risk of pneumonia in nursing home residents. A concentration of 0.23% PVP-I was showed rapid bactericidal activity and virucidal efficacy against *K. pneumoniae*, *S. pneumoniae*, severe acute respiratory syndrome associated coronavirus, Middle East respiratory syndrome associated coronavirus, H1N1 and rotavirus (Eggers et al., 2018). It could provide a protective oropharyngeal hygiene measure for individuals at high risk of exposure to oral and respiratory pathogens (Eggers et al., 2018). For ICU nursing, using 0.2% CHX gel three times a day to clean gingiva and dental plaque significantly decreases the colonization rate of oropharyngeal aerobic pathogens in artificially ventilated patients (Fourrier et al., 2005). A recent study showed application of oral care protocol based on the removal of secretions from the oral cavity with 0. 12% CHX gluconate solution for brushing could significantly reduce the risk of development of VAP and *S. aureus* infection (Galhardo et al., 2020) . In addition to the use of CHX, specific antibiotics against *P. gingivalis*, *F. nucleatum*, *A. actinomycetemcomitans or streptococci* (including *S. oralis*, *S. pyogenes*, *S. agalactiae*, *S. intermedius*, and *S. mitis*) are pointed out as a further direction in controlling respiratory infections, since these bacteria are reported to promote the colonization or virulence of respiratory pathogens (King et al., 2005; Pan et al., 2009; Li et al., 2014).

Utilization of probiotics is also proposed as a strategy to fight against respiratory infections. Multiple studies have demonstrated the negative effect of oral *lactobacilli, streptococci* on respiratory pathogens and the potential of these bacteria as probiotics in the future to combat various lung infections (Alexandre et al., 2014; Scoffield and Wu, 2015; Kang et al., 2017; Scoffield et al., 2017; Mahdi et al., 2019).

### Attentions in Dental Treatment

Patients with COPD have large amounts of mucous secretions (chronic bronchitis) with repeated coughing or dyspnea (emphysema) caused by airway destruction. If possible, it is recommended that during dental treatment, severely affected COPD patients be treated by sitting upright in dental chairs because they may experience difficulties in breathing while lying flat. At the same time, taking into account the important role of dental care and oral health knowledge in the prevention and treatment of COPD, attention should be paid to their oral hygiene guidance (Devlin, 2014).

Since an asthma attack could be triggered or exacerbated by a lot of risk factors, asthma patients should receive greater attention in dental treatment. It is suggested to assess the risk level of an asthma patient by a dental professional to decide whether the patient’s health is stable enough to proceed with treatment and make sure that the patient has his or her own rescue inhaler on hand and on the bracket table (Little et al., 2017). All dental operations as well as dental instruments and materials used during dental treatment should not trigger an asthma attack and prolonged supine positioning should be avoided (Khalifa et al., 2014; Harrington et al., 2016).

Although the number of reported cases of infection or respiratory symptoms caused by dental waterway pollution is limited, the American Dental Association requires that the routine dental treatment output water used in dental unit waterlines should meet the quality standard for drinking water (i.e., ≤500 CFU/ml of heterotrophic water bacteria) and it is recommended to use sterile saline or sterile water as a coolant or irrigant when performing surgical procedures (Kohn et al., 2003).

## Conclusions and Further Directions

The relationship between oral microecosystem and respiratory diseases have been proved by plenty of studies. On the one hand, indigenous oral microorganisms take a part in the occurrence and development of respiratory diseases via their pathogenicity and virulence factors when inhaled into respiratory tract or interact with respiratory pathogens. On the other hand, many respiratory pathogens are found to adapt to the fluctuational oral environment with the help of their biological characteristics and their interspecies interactions with oral residents. Although we are making continuous progresses on understanding the role of oral microecosystem in development of respiratory diseases, etiological evidence that relates respiratory diseases with oral microecosystem is still insufficient. There are a lot of aspects to be explored and studied, including: (1) the specific oral microorganisms related to different respiratory diseases, and the potential mechanisms involved in the pathogenic process; (2) the adaptation and colonization mechanisms of respiratory pathogens in oral microecosystem, influence factors and available intervention measures; (3) the specific oral health management and treatment measures for patients or susceptible populations of respiratory diseases; (4) the potential oral biomarkers or indexes such as the abundance of specific microbes to predict the progress of respiratory diseases.
